# In Silico and In Vitro Evaluation of the Molecular Mimicry of the SARS-CoV-2 Spike Protein by Common Short Constituent Sequences (cSCSs) in the Human Proteome: Toward Safer Epitope Design for Vaccine Development

**DOI:** 10.3390/vaccines12050539

**Published:** 2024-05-14

**Authors:** Yuya Mizuno, Wataru Nakasone, Morikazu Nakamura, Joji M. Otaki

**Affiliations:** 1 The BCPH Unit of Molecular Physiology, Department of Chemistry, Biology and Marine Science, Faculty of Science, University of the Ryukyus, Senbaru, Nishihara 903-0213, Okinawa, Japan; 2 Computer Science and Intelligent Systems Unit, Department of Engineering, Faculty of Engineering, University of the Ryukyus, Senbaru, Nishihara 903-0213, Okinawa, Japan

**Keywords:** SARS-CoV-2, spike, short constituent sequence, human proteome, epitope, autoantibody, autoimmune disease, vaccine development, molecular mimicry, immunological tolerance

## Abstract

Spike protein sequences in SARS-CoV-2 have been employed for vaccine epitopes, but many short constituent sequences (SCSs) in the spike protein are present in the human proteome, suggesting that some anti-spike antibodies induced by infection or vaccination may be autoantibodies against human proteins. To evaluate this possibility of “molecular mimicry” in silico and in vitro, we exhaustively identified common SCSs (cSCSs) found both in spike and human proteins bioinformatically. The commonality of SCSs between the two systems seemed to be coincidental, and only some cSCSs were likely to be relevant to potential self-epitopes based on three-dimensional information. Among three antibodies raised against cSCS-containing spike peptides, only the antibody against EPLDVL showed high affinity for the spike protein and reacted with an EPLDVL-containing peptide from the human unc-80 homolog protein. Western blot analysis revealed that this antibody also reacted with several human proteins expressed mainly in the small intestine, ovary, and stomach. Taken together, these results showed that most cSCSs are likely incapable of inducing autoantibodies but that at least EPLDVL functions as a self-epitope, suggesting a serious possibility of infection-induced or vaccine-induced autoantibodies in humans. High-risk cSCSs, including EPLDVL, should be excluded from vaccine epitopes to prevent potential autoimmune disorders.

## 1. Introduction

Since the outbreak of COVID-19 in December 2019, the development of therapies and vaccines based on available knowledge on this disease has been a major medical concern worldwide [1]. Basic research on COVID-19 and its pathogen, SARS-CoV-2, has progressed very quickly in the few years since the outbreak, leading to the prompt development of vaccines [2,3]. This rapid progress is hallmarked by prompt identification of the pathogen and its genome sequencing [4,5,6] and subsequent structural analyses of viral proteins [7,8,9,10]. One of the most important achievements in intervening medical technology against SARS-CoV-2 is the use of mRNA-based vaccines to lessen the risk of SARS-CoV-2 infection as well as the poor prognosis for patients with COVID-19 [11,12,13]. Nobel Prize-winning mRNA vaccines are designed to express the spike protein of SARS-CoV-2 in human muscles for the production of neutralizing antibodies. The efficacy of these vaccines has been reported to be very high, although some side effects have also been reported [14,15,16,17,18].

Despite this success, there appears to be a group of people who are infected after vaccination, partly because the immunized state induced by infection or vaccination does not last long, which necessitates repeated vaccination to prevent future infection and severe symptoms [19,20]. There are people who experience long-term symptoms, referred to as long COVID-19 patients [21,22,23]. These facts suggest that scientists do not yet fully understand the processes of SARS-CoV-2 infection, vaccination, and immune system activation, including the induction of anti-SARS-CoV-2 antibodies and immunological memory formation against SARS-CoV-2. Therefore, efforts to produce safer vaccines should continue based on a mechanistic understanding of the interactions between SARS-CoV-2 and humans.

Efficient antibody production depends on the proper recognition of viral epitopes, which are usually surface proteins on virions, by the immune system. The spike protein of the SARS-CoV-2 virion binds to the receptor angiotensin-converting enzyme 2 (ACE2) to initiate the infection process [7,8,9,10]. Current SARS-CoV-2 vaccines use spike protein sequences for epitopes. The important point in immunological recognition of viral proteins is that the human immune system must discriminate SARS-CoV-2 proteins (nonself) from its own proteins (self) as a result of complex processes involving regulatory T cells [24,25]. To be a functional epitope, a peptide should be presented by dendritic cells via MHC molecules to activate T cells and then B cells [26,27,28,29,30,31]. Peptide size held by MHC is restricted, and peptide size recognized by T-cell receptors and B-cell receptors is likely even smaller, probably as small as five amino acids in length [32,33,34].

Theoretically, the immune system should not produce antibodies against self-proteins simply because autoantibodies cause autoimmune disorders. Pathogens and parasites, such as viruses and bacteria, might have evolved to have sequences similar to those of humans to avoid immunological recognition. Such “molecular mimicry” has been argued to be an important driving force for viral evolution [35,36,37]. Alternatively, common sequences between pathogens and humans occur just coincidentally, especially when a sequence of interest is short (e.g., a stretch of sequence containing five amino acids). There is always a pure chance to encounter viral amino acid sequences that are identical to human sequences, which poses a potential risk of producing autoantibodies [37]. This unit of commonality may be as small as five amino acids in length.

There are many discussions on the possible contributions of molecular mimicry of pathogens to human autoimmune diseases [38,39,40,41,42,43,44], but a general conclusion has not yet been reached. The term “mimicry” in biology originally came from phylogenetically distant butterflies with similar wing color patterns to cheat their predators [45]. To general biologists, “mimicry” of pathogens implies the evolutionary consequences of pathogens cheating the host immune system. A classic example of molecular mimicry is probably the λ phage repressor, which structurally mimics LexA, leading to the cleavage of the λ phage repressor by RecA in the SOS response [46]. In immunology, “molecular mimicry” has been used for identifying sequence similarities between pathogens and the host regardless of evolutionary cheating, suggesting that such similarities may induce autoantibodies leading to autoimmune diseases. Many studies have reported that COVID-19 is associated with autoimmune responses [47,48,49,50,51,52,53,54,55,56,57,58,59,60,61,62,63,64]. Indeed, many spike epitopes of antibodies from COVID-19 patients have been identified [65,66,67,68,69,70,71,72,73,74,75,76], but whether these anti-spike antibodies cross-react with human proteins is unknown.

To understand how the human immune system discriminates SARS-CoV-2 proteins (nonself) from its own proteins (self), we have been using the concept of short constituent sequences (SCSs) of amino acids in proteins [77,78,79,80,81,82,83]. The idea of information extraction from protein amino acid sequences based on SCSs is as old as Chou and Fasman (1974) [84] and Garnier et al. (1978) [85]. In addition to ours, there are similar but independent approaches in the literature [86,87,88,89,90,91,92,93,94,95,96,97,98,99]. The number of amino acids in an SCS unit can vary, but we primarily use five amino acids (5-aa SCSs) [77,78,79,80,81,82,83]. The 5-aa window is technically convenient because there are only 20^5^ combinations of 5-aa SCSs (also called pentats in our system but called pentapeptides or pentamers in others), which is not computationally demanding. It is possible to comprehensively cover all informatically possible epitopes and all human proteins. The 5-aa window is also biologically feasible because the smallest unit of protein–protein interactions is often five amino acids [32,33,34]. Antibodies probably recognize epitopes with five amino acids at a minimum. On the other hand, longer windows, such as 8 aa or more, have been used, for example, in a different study [100] based on the length of peptides presented in MHC class I [101]. This is acceptable as a search condition, but in that case, shorter ones will certainly be missed. On the contrary, our search for 5-aa SCSs can identify not only 5-aa SCSs but also longer ones simultaneously as consecutive 5-aa SCSs. Previously, our protein studies demonstrated that SCS analyses are useful for identifying protein characteristics, including secondary structures [81,82] and functional sites [79,80], and human-specific proteins [83]. Alignment-independent SCS analyses are useful for identifying commonalities (instead of similarities) that have been overlooked by conventional alignment-dependent approaches.

Applying the SCS concept to SARS-CoV-2, we have previously reported that the SARS-CoV-2 spike protein is composed mainly of self SCSs (which are present both in SARS-CoV-2 and humans) but contains some nonself SCSs (which are present in SARS-CoV-2 but not in humans) [62] and that the Omicron variant of SARS-CoV-2 accumulates nonself mutations that are more antigenic in the RBD of the spike protein [102]. Similar in silico studies on molecular mimicry of SARS-CoV-2 have been published by different groups. For example, in one study, pentapeptides (i.e., 5-aa SCSs) were computationally searched for in the protein pool containing human oogenesis-related proteins, resulting in the identification of 41 cSCSs [59]. Most of them were found in the experimentally validated Immune Epitope Database (IEDB) [59]. In another study, specific 8-mer/9-mer peptides (i.e., 8-aa and 9-aa SCSs) from SARS-CoV-2 were found to be present in four human proteins [100]. These studies demonstrated that SCS or similar approaches to proteins are useful for understanding the protein amino acid sequences of SARS-CoV-2 in relation to the human proteome bioinformatically.

However, there are limitations to these in silico studies. The occurrence of self SCSs does not immediately imply a risk of autoantibody production in humans. Such self SCSs may be buried in depth within a protein molecule, in which case such sequences cannot function as epitopes. Human proteins with potential autoepitopes may be present within cells and may not be exposed in the extracellular milieu, in which case such sequences in human proteins cannot be a threat to human health. Only when self SCSs are located in extracellular proteins or extracellular portions of membrane proteins and are located on the surface of proteins with relatively high accessibility in humans may such autoepitopes threaten human health, leading to potential autoimmune disorders. In other words, even if anti-spike antibodies are produced against sequences that are also present in human proteins, such antibodies may not be able to recognize human proteins. Furthermore, B-cell clones that produce such autoantibodies may be forbidden even before SARS-CoV-2 infection by regulatory T cells, in which case autoantibodies will never be produced. However, the levels of self/nonself definitions are not absolute for the immune system. When the human body is exposed to a massive number of autoepitopes at the time of infection or vaccination, previously forbidden clones may be produced. Overall, one may conclude that although a bioinformatics approach reveals a potential risk of autoimmunity, it does not necessarily present a real risk of autoimmunity during infection or vaccination. However, at this point, current information is not sufficient to evaluate the effect of molecular mimicry of SARS-CoV-2 on human health. In addition to in silico studies, in vitro studies are required to evaluate the potential contribution of molecular mimicry to autoimmune disorders.

As mentioned above, we believe that, in general, vaccine epitopes should not contain self-sequences that occur in the human proteome for the sake of safety. In this sense, a self-sequence check for epitope sequences in vaccines should be performed before vaccine production. In reality, most SARS-CoV-2 vaccines use entire spike protein sequences for immunological epitopes without paying attention to the abundant self SCSs in the spike protein. Self-sequence checks should be performed not only in silico but also in vitro. Because there are many self SCSs in the spike protein [62], it is interesting to know which human proteins have these SCSs. Here, we introduce the term “common SCSs (cSCSs)” for self SCSs for easier understanding, indicating that SCSs occur both in the SARS-CoV-2 spike protein and the human proteome.

In this study, the potential contribution of molecular mimicry (or molecular commonalities, to be more precise) to the autoimmune response was examined systematically. We first identified cSCSs between the SARS-CoV-2 spike protein and the human proteome bioinformatically. Among these cSCSs, six cSCSs in six human proteins were obtained as candidates for autoepitopes. They were mostly located in three regions within the S1 portion of the spike protein. Polyclonal antibodies were produced using these three regions as epitopes. We demonstrated that one of these anti-spike antibodies cross-reacted with human proteins in vitro. We believe that this study will aid in rational and safer epitope design for vaccine development in general in the future.

## 2. Materials and Methods

### 2.1. Basic Bioinformatics

We previously identified self SCSs and nonself SCSs in the SARS-CoV-2 spike protein in reference to the human proteome [62]. Briefly, the reference genome sequence data (RefSeq) of humans and SARS-CoV-2 were obtained from NCBI (the National Center for Biotechnology Information, Bethesda, MD, USA) from the following URLs: https://www.ncbi.nlm.nih.gov/datasets/genomes/?acc=GCF_000001405.39 (NCBI RefSeq assembly of *Homo sapiens*; accessed on 14 November 2022) and https://www.ncbi.nlm.nih.gov/nuccore/1798174254 (severe acute respiratory syndrome coronavirus 2 isolate Wuhan-Hu-1, complete genome; NC_045512.2; accessed on 13 November 2022). The following operation was based on five amino acid windows sliding one amino acid at a time.

To obtain information on cSCSs in human proteins, we processed the sequence data above informatically. All SCSs that occurred in the spike protein were listed in the base dictionary. According to this base dictionary, the spike protein sequence was transformed into an scsVector, in which each vector component corresponding to each SCS in the base dictionary was expressed as 1 (present) or 0 (not present). The same operation was performed for each human protein in accordance with the base dictionary of the spike protein. The scsVectors of spike and human proteins were compared to each other. As a result, an SCS species and its number of occurrences were recorded in the list of human proteins with cSCSs (Supplementary File S1, “SARS-CoV-2 Spike vs. Human Original Text”). In this list, human proteins were listed from the highest number of occurrences of cSCSs to the lowest number, excluding zero. The list was imported into Microsoft Excel (Supplementary File S2, “Spike vs. Human Excel #1”).

The list of human proteins contained many isoforms, which were considered single proteins and thus bundled together. When two or more isoforms were present, the average number of cSCSs was calculated. Similarly, two or more precursors and preproteins were treated as a single protein. The number of amino acids was also calculated as average values for the isoforms, precursors, and preproteins. We calculated the “cSCS frequency” in a given protein as the number of cSCSs divided by the number of amino acids in that protein. To do so, cSCS frequencies were calculated first for each isoform, precursor, or preprotein, and these numbers were averaged. We made a rank-frequency plot to visually examine a potential stochastic power law distribution. One of the power law distribution patterns is called Zipf’s law. This is an empirical scaling law that may emerge due to a conflict between two systems [103,104,105]. Speakers and hearers are two conflicting systems that were originally proposed by Zipf (1949) [103]. When negative linearity is observed in a logarithmic rank-frequency plot over a few orders of magnitude with the exponent close to 1, it is customary to mention that the distribution follows Zipf’s law [103,104,105]. We also made scatter plots between the number of amino acids in a protein (*x*-axis) and the number of SCSs in a protein (*y*-axis). The input and output data were recorded in Microsoft Excel (Supplementary File S3, “Spike vs. Human Excel #2”). In silico analyses after the data transfer to Microsoft Excel were performed manually or using codes for Microsoft Excel (Supplementary File S4, “Excel Programming for Informatics”).

### 2.2. Functional Classification of cSCS-Containing Human Proteins

We used Gene Ontology (GO) to functionally classify the cSCS-containing human proteins at the URL https://geneontology.org (accessed on 17 June 2022). All cSCS-containing human proteins were classified into a function-based categorization of GO. To this end, we first downloaded 31 files for functional classification (biology_process) of all human proteins from AmiGO 2 (AmiGO 2: Drill-down Browser (geneontology.org)) of GO to Microsoft Excel. These files were compared to the list of cSCS-containing proteins, and their functional categorization was determined. A given protein may be categorized into two or more GO categories, according to our analysis. We calculated ratios of the number of cSCS-containing proteins to the number of all proteins in each category.

### 2.3. cSCSs in Human Proteins

We basically assumed that human proteins with higher cSCS frequencies were more likely to be functional (cross-reactive) epitopes simply because there were more cSCSs in a protein chain. Multiple cSCSs in a human protein often indicate the possibility that they are consecutive (fusion) cSCSs that form a cSCS cluster of six or more amino acid residues. To extract proteins with multiple cSCSs, we constructed a rank list of human proteins according to the number of cSCSs. Human proteins containing five or more cSCSs were examined for cSCSs and their positions in the three-dimensional structure of proteins. We referred to the Protein Data Bank (PDB) at the URL https://www.rcsb.org (accessed on 20 November 2022). We visually examined the three-dimensional structures of proteins using the built-in 1D–3D View function of PDB and PyMol at the URL https://pymol.org (accessed on 20 November 2022) to clarify whether a given cSCS was located on the surface or depth of that protein molecule. Furthermore, we obtained solvent accessibility from the outside of a protein molecule as a quantitative evaluation of the three-dimensional location of the cSCS using the SPPIDER (Solvent accessibility-based Protein–Protein Interface iDEntification and Recognition) tool at the URL https://sppider.cchmc.org (accessed on 29 January 2023). To do so, we used “Prediction of interaction sites using an unbound protein 3D structure”, which refers to 3D information from PDB-ID (Method I). When accessibility could not be obtained via this method, we also used “Sequence-based prediction of interaction sites (Experimental)”, which refers to the prediction of 3D structures based on amino acid sequence information (Method II). Accessibility was evaluated as a score ranging from 0 (least accessible) to 9 (most accessible). Furthermore, we examined whether cSCS-containing proteins or their portions were extracellular using information shown in “Subcellular Location” in UniProt (Universal Protein Resource) at the URL https://www.uniprot.org (accessed on 19 November 2022).

We also hypothesized that cytokines may have a greater chance of having cross-reactive epitopes of cSCSs because COVID-19 is characterized by a cytokine storm [106,107,108,109]. From the original list, we extracted proteins that had annotations of at least one of the following words: interleukin (IL), interferon (IF), tumor necrosis factor (TNF), or tumor growth factor (TGF). Subsequent procedures were the same as those for the other proteins described above. We focused on cytokines containing three or more cSCSs.

### 2.4. Peptide Synthesis and Conjugation

Antigenic peptides for anti-spike (SARS-CoV-2) antibodies were designed to include cSCSs that were shown to be present in extracellular proteins or the extracellular domain of membrane proteins with reasonable solvent accessibility. We designed three peptides (Peptides 1, 2, and 3), with sequences taken from spike S1 as follows: Peptide 1 was CGWIFGTTLDSKTQ (14 aa), in which the N-terminal C was added for carrier conjugation (also in Peptide 2). This peptide contains a cSCS (GTTLD) shared with human tenurin-4. Peptide 2 was CALEPLVDLPIGINITRFQTLLALHR (26 aa). This peptide contains two consecutive cSCSs (EPLVD and PLVDL) shared with the human unc-80 protein and one cSCS (TLLAL) shared with the human ryanodine receptors 1, 2, and 3. Peptide 3 was SPTKLNDLCFTNV (13 aa). This peptide contains three consecutive cSCSs (KLNDL, LNDLC, and NDLCF) shared with human IL7. The presence of antibodies against these epitopes in COVID-19 patients was checked with the Immune Epitope Database (IEDB) at the URL https://www.iedb.org (accessed on 10 March 2024) [110,111,112]. The peptides above were synthesized using the Fmoc solid-phase peptide synthesis method by Cosmo Bio (Tokyo, Japan). An azido group was added to the amino terminus of Peptide 3 by click chemistry. The purity of these peptides was examined with HPLC and TOF-MS (Shimadzu AXIMA Confidence 2.9.3, Kyoto, Japan) at 220 nm, confirming that the percentage of the LC peak area was at least 73% and that the experimentally obtained molecular weight was close enough to the theoretical molecular weight.

Before immunization of rabbits, the SH group of the cysteine residue in Peptides 1 and 2 above was conjugated with the amino group of the KLH (keyhole limpet hemocyanin) carrier protein via an MBS (*m*-maleimidobenzoyl-*N*-hydroxysuccinimide ester) linker. Conjugation was confirmed by the Ellman method at 412 nm as a decrease in the number of free SH groups after successful conjugation. Peptide 3 was conjugated with KLH via an N-terminal azido group.

For competitive inhibitor assays, we designed seven potential cross-reactive peptides, with the sequences obtained from human proteins. These peptides included a single cSCS, either EPLDV or TLLAL, as follows: Peptide U, RTEPLVDLES (10 aa), from unc-80; Peptide RY, STETLKTLLALGG (13 aa), from the ryanodine receptor; Peptide M1, PYPMSKLVTLLALGG (15 aa), from multimerin-2; Peptide M2, KEAEPLVDIRVTGPVP (16 aa), from multimerin-2; Peptide C, LIAGAIIGTLLALALI (16 aa), from the coxsackievirus and adenovirus receptor; Peptide RA, IPEGDPENTLLALKKP (16 aa), from ras GTPase-activating-like protein IQGAP2; and Peptide T, KQDGKSRGTLLALEGP (16 aa), from thrombospondin-2. These peptides were synthesized by Cosmo Bio (Tokyo, Japan) as the antigenic peptides described above.

### 2.5. Antibodies

Using the synthetic peptides described above, rabbit polyclonal antibodies were raised in the laboratory of Cosmo Bio (Tokyo, Japan) using the Fast Antibody Plus service. A rabbit (Japanese White) was immunized with a KLH-conjugated peptide at days 0, 14, 28, and 42. The first injection on day 0 involved the administration of 400 μg, and the subsequent injections involved the administration of 200 μg. On days 0 and 35, blood samples (2 mL) were obtained to assess antibody production. All blood samples (40–45 mL) were obtained on day 56. Blood serum samples (10 mL) were subjected to peptide column chromatography for affinity purification. Purified antigen was stored at 4 °C before use with ProClin 300 (catalog number 48914-U, Sigma-Aldrich, Merck, Rahway, NJ, USA) at 15 ppm. Antibody concentrations were measured with ELISA using a secondary antibody (anti-rabbit IgG) conjugated with alkaline phosphatase at 405 nm in the laboratory of Cosmo Bio. The final concentrations of antibodies against Peptides 1, 2, and 3 were 0.28, 1.51, and 0.46 mg/mL, respectively.

In addition, we purchased a protein G-affinity-purified anti-spike (SARS-CoV-2) rabbit polyclonal IgG antibody (catalog number PAB31705) from Abnova (Taipei, Taiwan). This antibody was raised against the entire SARS-CoV-2 spike protein as an epitope. This antibody was supplied at a concentration of 2 mg/mL from the manufacturer. Additionally, an IL-7 affinity-purified anti-human IL-7 rabbit polyclonal IgG antibody (catalog number 500-P27) was purchased from PeproTech (Thermo Fisher Scientific, Waltham, MA, USA). This antibody was raised against human IL-7 (153 aa) expressed in *Escherichia coli* and supplied as a lyophilized powder. The solution was reconstituted in ultrapure water to a concentration of 0.1 mg/mL.

### 2.6. ELISA (Enzyme-Linked Immunosorbent Assay)

To test the affinity of the antibodies for the spike protein, we used the Human SARS-CoV-2 Spike (Trimer) IgG ELISA Kit (catalog number BMS2325; Invitrogen, Thermo Fisher Scientific) in accordance with the manufacturer’s instructions. In this kit, the spike protein is immobilized on the surface of a microplate. After the test sample was added, an anti-IgG antibody conjugated with HRP (horse radish peroxidase) via biotin was added. The color was developed by adding TMB (tetramethylbenzidine) as a substrate chromogen for HRP, which was then stopped by adding the stop solution. The color of the solution at 450 nm was measured by a Corona Electric Multigrating Microplate Reader SH-9000Lab (Hitachinaka, Ibaraki, Japan). This kit was also used to test the competitive binding of synthetic peptides containing the cSCSs of interest. ELISA was performed in triplicate for a given sample throughout this paper.

To test the ability of antibodies to inhibit the spike-ACE2 interaction (i.e., to test the neutralizing ability of antibodies), we used the SARS-CoV-2 Spike S1/ACE2 Binding Inhibitor Screening Kit (Catalog Number COV-K01; Cosmo Bio, Tokyo, Japan) according to the manufacturer’s instructions. Ni-NTA HisSorb Plates (96 microwell plates) (Catalog Number 35061; QIAGEN, Hilden, Germany) were used to immobilize the ACE2-His tag on the surface of the plates. Spike (S1) was tagged with Fc, which was recognized by an anti-mouse IgG antibody conjugated with HRP. TMB was used as a substrate for HRP. This kit has been developed based on the molecular mechanisms of the spike-ACE2 interaction [113,114,115] and its inhibition [116].

### 2.7. Western Blot Analysis

To test the cross-reactivity of rabbit antibodies raised against spike peptides containing cSCSs, we performed Western blot analysis using a premade Western blot membrane, INSTA-Blot Human Tissues (Catalog Number NBP2-30113; Novus Biologicals, Centennial, CO, USA). According to the manufacturer’s specifications, in this PDVF membrane, each lane contained 20 μg of human protein lysate, as measured by the Bradford assay. Blotting of proteins on the PVDF membrane was performed electrically after SDS-PAGE (Tris-glycine gel). Tissue samples were prepared from human individuals without known diseases by the manufacturer. The use of commercial samples does not require institutional review or informed consent.

We obtained a PVDF membrane stained with amido black to confirm the presence of the blotted proteins. The membrane was first immersed in 100% methanol and then in PBS-T (0.1% Tween, Kanto Chemical, Tokyo, Japan) twice. Amido black was removed with 5% acetate for 1 min twice and then washed with ultrapure water for 10 min twice [117]. The membrane was then incubated in PBS-T with 5% skim milk (Morinaga, Tokyo, Japan) for 1 h. Then, the membrane was incubated with rabbit anti-cSCS (spike) antibodies against cSCS peptides in PBS-T for 1 h at room temperature or overnight at 4 °C. The membrane was then washed in PBS-T three times and incubated for 1 h at room temperature with a secondary anti-IgG antibody conjugated with HRP, which was obtained from the Human SARS-CoV-2 Spike (Trimer) IgG ELISA Kit (Thermo Fisher Scientific). The primary and secondary antibodies were diluted with a 1:1 mixture of PBS-T and Can Get Signal Immunoreaction Enhancer Solution (catalog number NKB-101T; TOYOBO; Osaka, Japan). After being washed three times, the membrane was immersed for 20 min in Pierce 1-Step Ultra TMB-Blotting Solution (catalog number 37574; Thermo Fisher Scientific) for colorimetric detection. The developed membrane was scanned at 600 dpi for image acquisition, and the bound antibodies were then removed using EzReprobe (catalog number WSE-7240; ATTO, Tokyo, Japan) for subsequent tests with additional antibodies on the same membrane.

## 3. Results

### 3.1. Rationales and Strategies

**Figure 1:**
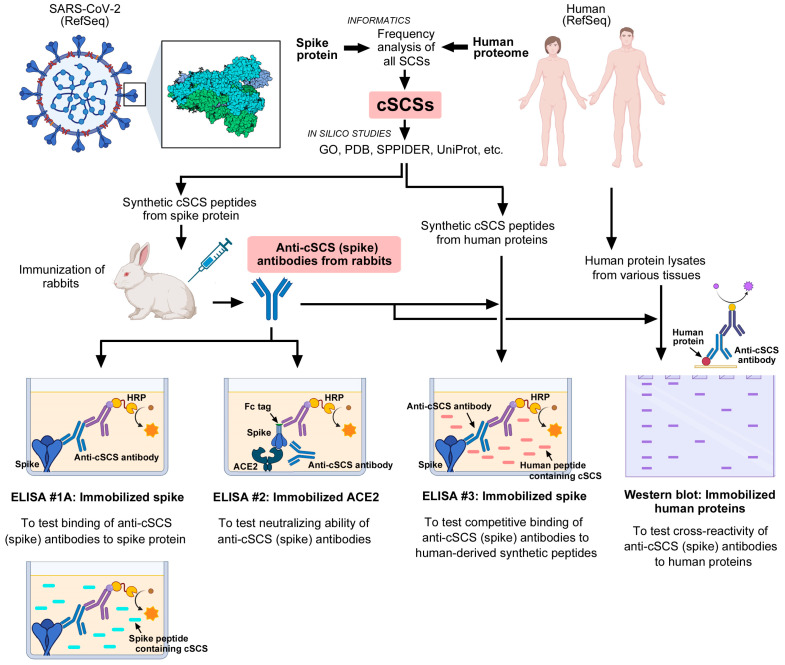
Overview of the present study. Common short constituent sequences (cSCSs) between the SARS-CoV-2 spike protein and the human proteome were identified informatically. Based on these cSCSs, anti-cSCS (spike) antibodies were developed against synthetic cSCS peptides. Anti-cSCS (spike) antibodies were tested by ELISA and Western blot analyses. This figure was created with BioRender.com and Adobe Photoshop Elements Version 14.

The overall strategy of this study is shown in Figure 1. This study is based on the simple concept that some short constituent sequences (SCSs) of SARS-CoV-2 may occur in humans, and the co-occurrence of such sequences may induce autoantibodies in humans. Considering that the number of different stretches of five amino acid (5-aa) sequences is limited to 20^5^ (=3.2 million), it is possible to comprehensively count and map all possible types of SCSs in a given proteome. An SCS that occurs both in the SARS-CoV-2 spike protein and in the human proteome is called the common SCS (cSCS). Because identical five amino acid sequences occur in SARS-CoV-2 and humans, this analysis is not based on similarity or homology but rather on commonality. We employed the concept of cSCS frequencies (or occurrences or counts) between the SARS-CoV-2 spike protein and the human proteome. The identified cSCSs were then subjected to further bioinformatics to determine the statistical characteristics of the cSCSs. To determine whether the bioinformatically identified cSCSs function as cross-reacting epitopes for autoantibodies in vitro, we obtained polyclonal anti-cSCS (spike) antibodies from rabbits, and the antibodies were characterized via ELISA and Western blot analyses.

### 3.2. Informatical Characterization of Human Proteins Containing cSCSs

**Figure 2:**
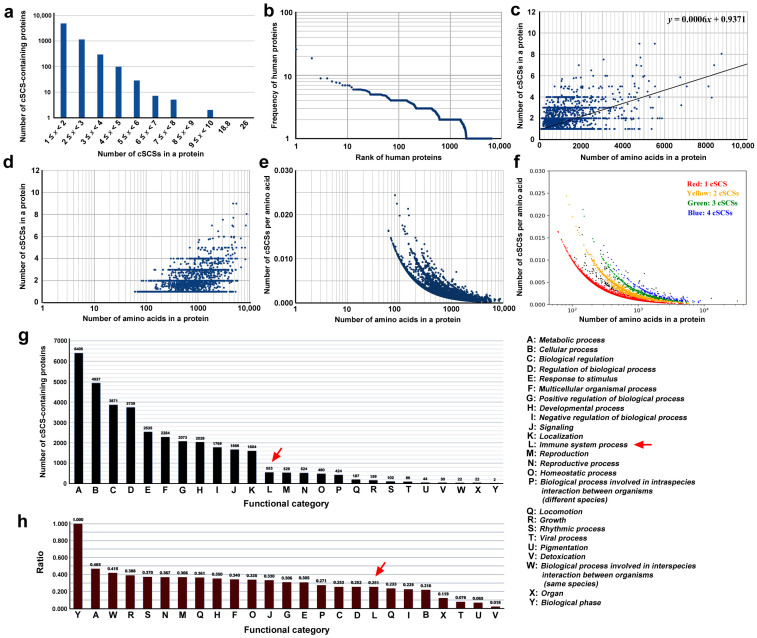
Informatical analyses of cSCS-containing human proteins. (**a**) Histogram of the number of proteins in response to the number of cSCSs in a protein. (**b**) Rank-frequency plot. Human proteins are ranked in the order of the number of cSCSs in a protein. (**c**) Scatter plot of the number of cSCSs in human proteins in response to the number of amino acids in a protein (protein length). A linear fit (*y* = 0.0006*x* + 0.9371) is shown. (**d**) The same scatter plot as (**c**) except for the log scale on the *y*-axis. (**e**) Scatter plot of the number of cSCSs per amino acid in response to the number of amino acids in a protein. The *y*-axis is shown on a log scale. (**f**) The same scatter plot as (**e**), except for the assigned colors. (**g**) Functional categorization of cSCS-containing human proteins in accordance with the GO assignments. The functional categories A–Y are shown on the right. The red arrow indicates the immune system process. (**h**) Ratio of the number of cSCS-containing proteins to all the proteins in a functional category. The red arrow indicates the immune system process.

We have previously identified self SCSs and nonself SCSs in the SARS-CoV-2 spike protein informatically [62]. Here, we identified common SCSs (cSCSs) in the human proteome based on the human reference genome. There were 6405 human proteins that contained cSCSs shared with the SARS-CoV-2 spike protein (Supplementary Files S1–S3). The highest number of cSCSs in a protein was observed for titin (26 cSCSs), followed by mucin-16 (18.8 cSCSs). These high numbers of cSCSs in titin and musin-16 were likely because these proteins are very long. The histogram of the number of proteins in response to the number of cSCSs in a protein showed that a group of proteins that contained just one cSCS per protein was the most frequent (Figure 2a). The rank-frequency plot of all 6405 cSCS-containing proteins revealed a scale-free nature in the *x*-axis (rank) in a few orders of magnitude, although the *y*-axis (the number of cSCSs per protein) was limited in two orders and its exponent was not close to 1 (Figure 2b). Thus, it cannot be said that this graph follows Zipf’s law, but it would show a nearly linear distribution following a stochastic power law distribution of cSCSs in the human proteome if the number of samples were amplified. Furthermore, there was no particular biological feature in this distribution. These results suggest that the proteome-wide incorporation of cSCSs into human proteins is mostly a process of probability and not of functional biological evolution.

To further understand the relationship between the number of cSCSs in a protein and protein length, a scatter plot was made, excluding titin (31,910 aa) and mucin-16 (12,872 aa) as outliers (Figure 2c,d). The linear fit showed an equation of *y* = 0.0006*x* + 0.9317, and the Pearson correlation coefficient *r* was 0.59, indicating that there was a weak relationship between these two factors. When the cSCS frequency (per amino acid) was used on the *y*-axis and both axes were shown on logarithmic scales, again excluding titin and musin-16, at least four different lines emerged (Figure 2e). These four lines corresponded to different numbers of cSCSs in a protein (Figure 2f). Overall, these plots revealed that the cSCS frequency (density) was greater for shorter proteins than for longer proteins. This is probably because there are more repeat sequences that do not contain any cSCS in longer proteins.

Human proteins containing cSCSs shared with the SARS-CoV-2 spike protein were found in all 25 functional categories given by GO (Figure 2g). The most enriched category was “metabolic process”, followed by “cellular process” and “biological regulation”. “Immune system process” was ranked 12th. Because the number of proteins in each GO category varied, the *y*-axis was transferred to the ratio of the cSCS-containing proteins to the entire proteins in a category by dividing the number of cSCS-containing proteins by the number of all proteins in that category. The cSCS-containing proteins were unlikely to be biased very much to a particular function (Figure 2h). The highest category was “biological phase”, but it contained only two proteins. The category “immune system process” was not particularly conspicuous in this graph. These results suggest that most cSCSs are unlikely to be produced through evolutionary interactions between SARS-CoV-2 and humans, but they occur just coincidentally without such interactions.

### 3.3. Molecular Characterization of the Human Proteins Containing cSCSs In Silico

Among the 6405 human proteins containing cSCSs shared with the spike protein, there were 75 proteins that contained cSCSs five times or more. Among these proteins, there were 20 that were present in the extracellular environment at least partially, as follows: ryanodine receptor 3, protein unc-80 homolog, ryanodine receptor 1, basement membrane-specific heparan sulfate proteoglycan core protein, ryanodine receptor 2, protocadherin Fat 4, apolipoprotein B-100, amiloride-sensitive sodium channel subunit alpha, teneurin-4, neural cell adhesion molecule L1-like protein, otogelin, musin-19, reelin, unconventional myosin-XVI, cytoplasmic dynein 1 heavy chain 1, musin-16, nesprin-2, obscurin, coiled-coil domain-containing protein 168, and mucin-3A. Among the cSCSs that were located in these proteins, 41 species of cSCSs in the following 17 human proteins were located on the surface of spike proteins: ryanodine receptor 3 (FVSGN, TLLAL, and SNNLD), protein unc-80 homolog (ECDIP, EPLVD, PLVDL, AGAAA, and LPDPS), ryanodine receptor 1 (GAGAA, SLLIV, TLLAL, and AGAAA), ryanodine receptor 2 (TLLAL), protocadherin Fat 4 (LVRDL and TSALL), apolipoprotein B-100 (SSTAS), teneurin-4 (GTTLD and RLFRK), neural cell adhesion molecule L1-like protein (QPTES and PSKPS), otogelin (PVLPF), musin-19 (PCSFG and CSFGG), reelin (TTLDS, NATRF, ASQSI, and EPQII), unconventional myosin-XVI (TNLVK), cytoplasmic dynein 1 heavy chain 1 (WFHAI, VRDLP, and LFNKV), musin-16 (STEKS, TTLDS, TLDSK, DISTE, SLGAE, PDPSK, SLSST, LSSTA, and SSTAS), nesprin-2 (DSFKE), coiled-coil domain-containing protein 168 (FPLQS, KIQDS, and DSLSS), and mucin-3A (TAGAA).

**Figure 3:**
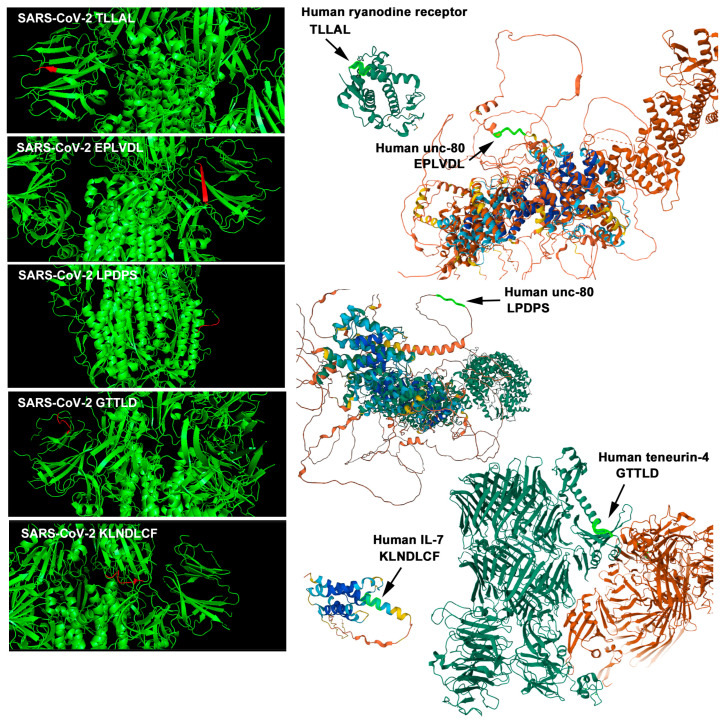
Three-dimensional structures of the cSCS regions of the SARS-CoV-2 spike protein (**left**) and human proteins (**right**). The cSCSs in the spike protein are shown in red, and those in human proteins are shown in light green (arrows).

Furthermore, among these 17 proteins, the locations of cSCSs were examined visually with the help of 3D structural images. The following six species of cSCSs were detected on the surface of six human proteins: ryanodine receptor 3 (TLLAL), protein unc-80 homolog (EPLVD, PLVDL, and LPDPS), ryanodine receptor 1 (TLLAL), ryanodine receptor 2 (TLLAL), teneurin-4 (GTTLD), and neural cell adhesion molecule L1-like protein (PSKPS) (Figure 3). The data are compiled in Table 1. Their accessibility scores were also examined (Table 1), confirming the results of the visual inspection.

Because smaller proteins appeared to have a greater density of cSCSs than longer proteins (Figure 2c–f) and because COVID-19 has often been associated with cytokine storms [101,102,103,104], we conducted searches for cytokines containing cSCSs. There were 50 cytokines containing at least one cSCS. Among them, only IL-7 contained three or more cSCSs. IL-7 contained KLNDL, LNDLC, NDLCF, and LVLLP. The first three cSCSs were consecutive and were not located on the surface of the spike protein but were found on the surface of IL-7. The last one, LVLLP, was not shown in 3D structures, likely because this portion of IL-7 is intrinsically disordered. Accessibility scores were also determined for these cSCSs (Table 1).

**Figure 4:**
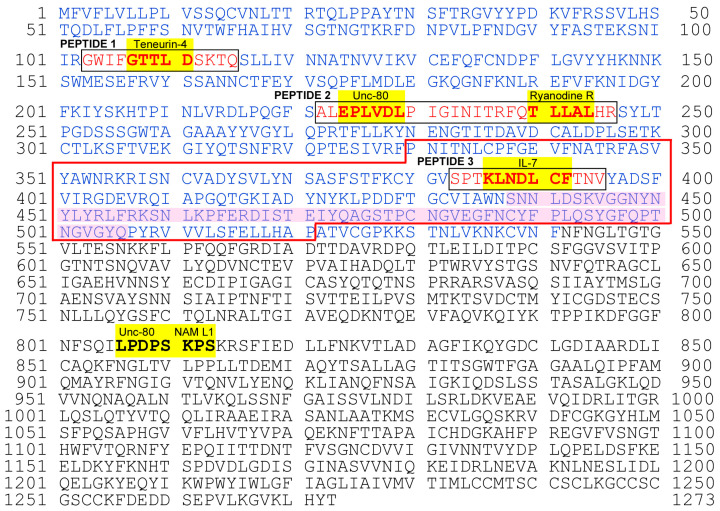
Complete amino acid sequence of the SARS-CoV-2 spike protein showing cSCSs and peptide epitopes for anti-cSCS (spike) antibodies. The entire sequence was obtained from NC_045512.2 (RefSeq). Amino acid residues in blue indicate the S1 domain. cSCSs are shown in bold and highlighted in yellow with the names of their corresponding human proteins. Peptides 1, 2, and 3 are boxed and shown in red. The receptor-binding domain (RBD) is enclosed in red lines, with the receptor-binding motif (RBM) shaded in pink. N-linked glycosylation sites are located at 603, 616, 657, 709, 717, 801, 1074, 1098, 1134, 1158, 1173, and 1194.

Therefore, we decided to focus on the TLLAL of ryanodine receptors 1, 2, and 3; EPLVD and PLVDL of the protein unc-80 homolog; GTTLD from teneurin-4; and KLNDL, LNDLC, and NDLCF of IL-7 for subsequent in vitro analyses (Figure 4). We excluded LPDPS from unc-80 and PSKPS from neural adhesion molecule L1-like protein from further analysis because they were present in S2 (Figure 4). Peptides 1, 2, and 3 for epitopes were designed from spike S1 sequences containing cSCSs (Figure 4). Among these cSCSs, those of IL-7 were present in the middle of the receptor-binding domain (RBD), whereas those of ryanodine receptors, the unc-80 homolog, and teneurin-4 were present outside the RBD. There were 12 N-linked glycosylation sites in the spike protein, but none of them were located in these peptides.

### 3.4. ELISA for Anti-cSCS Antibodies

**Figure 5:**
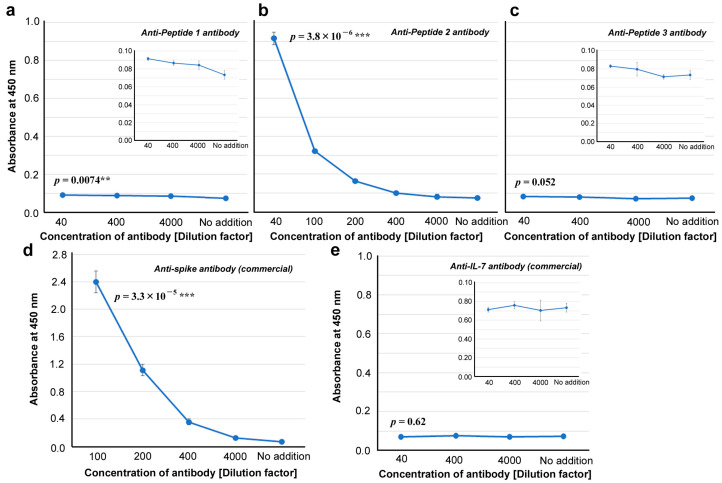
ELISA for antibody binding to spike protein (ELISA #1A in Figure 1). The indicated *p*-values were obtained by two-sided unpaired Student’s *t* tests between the samples with the least dilution factor and the samples with “no addition” of the antibody (control). The error bars indicate the standard deviation. Asterisks indicate statistical significance (**: *p* < 0.01, ***: *p* < 0.001). (**a**) Anti-Peptide 1 antibody. (**b**) Anti-Peptide 2 antibody. (**c**) Anti-Peptide 3 antibody. (**d**) Anti-spike antibody (commercial). (**e**) Anti-IL-7 antibody (commercial).

We successfully synthesized three peptides, Peptides 1, 2, and 3, and obtained three polyclonal anti-cSCS antibodies from rabbits using a standard protocol. The ELISA results showed that among the three anti-cSCS antibodies, the anti-Peptide 1 antibody (*p* = 0.0074) (Figure 5a) and anti-Peptide 2 antibody (*p* = 3.8 × 10^−6^) (Figure 5b) showed significantly greater absorbance than the “no addition” of the antibody. However, only the anti-Peptide 2 antibody seemed to be immunologically significant. The anti-Peptide 3 antibody did not show a statistically significant difference (*p* = 0.052). The commercial anti-spike antibody also showed a statistically significant difference (*p* = 3.3 × 10^−5^), indicating high affinity for the spike protein (Figure 5d). A commercially available anti-IL-7 antibody did not show any significant difference (*p* = 0.62) (Figure 5e).

**Figure 6:**
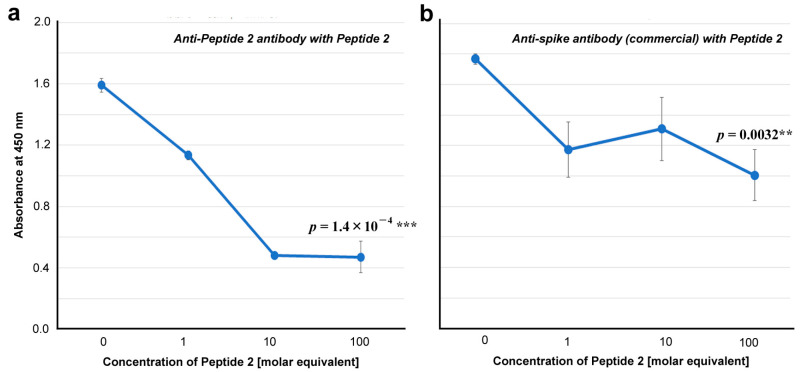
ELISA for antibody binding to the spike protein in the presence of Peptide 2 (ELISA #1B in Figure 1). The indicated *p*-values were obtained via two-sided unpaired Student’s *t* tests between the samples with the highest concentration tested (100 molar equivalent) and the samples with “no addition” of Peptide 2 (0 molar equivalent). The error bars indicate the standard deviation. Asterisks indicate statistical significance (**: *p* < 0.01, ***: *p* < 0.001). The molar equivalent was calculated by assuming that the molecular weight of IgG is 150 kDa. The molecular weight of Peptide 2 is 2917.5 Da. (**a**) Anti-Peptide 2 antibody in the presence of Peptide 2. (**b**) Anti-spike antibody (commercial) in the presence of Peptide 2.

To further test the specificity of the anti-Peptide 2 antibody against Peptide 2, we examined whether the addition of Peptide 2 competitively inhibited the interaction of the anti-Peptide 2 antibody with the spike protein. As expected, the addition of Peptide 2 significantly decreased the absorbance (*p* = 1.4 × 10^−4^), indicating the competitive inhibition and specificity of this antibody to the Peptide-2 sequence both in spike and Peptide 2 (Figure 6a). A commercially available anti-spike antibody was also affected by the addition of Peptide 2 (*p* = 0.0032), suggesting that a portion of this polyclonal anti-spike antibody interacts with the Peptide 2 sequence in the spike protein (Figure 6b).

**Figure 7:**
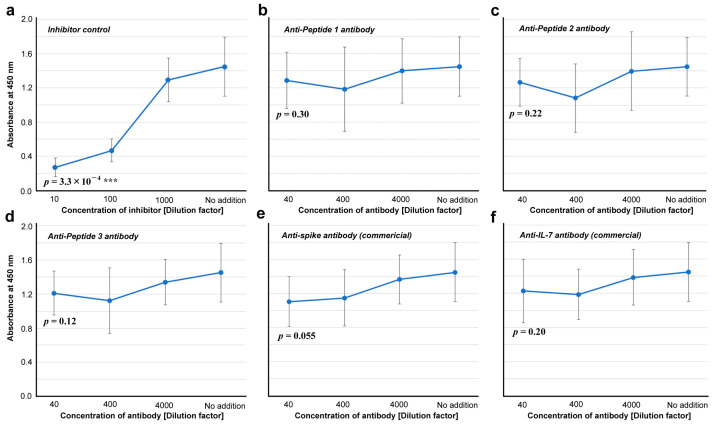
Inhibitory assay for the spike-ACE2 interaction (ELISA #2 in Figure 1). The indicated *p*-values were obtained by two-sided unpaired Student’s *t* tests between the samples with the least dilution factor and the samples with “no addition” of a potential inhibitor (control). The error bars indicate the standard deviation. Asterisks indicate statistical significance (***: *p* < 0.001). (**a**) Inhibitor control (ε-poly-L-lysine). (**b**) Anti-Peptide 1 antibody. (**c**) Anti-Peptide 2 antibody. (**d**) Anti-Peptide 3 antibody. (**e**) Anti-spike antibody (commercial). (**f**) Anti-IL-7 antibody.

We then tested whether these antibodies are capable of inhibiting ACE2 binding to the spike protein in vitro. Whereas an inhibitor control (ε-poly-L-lysine) showed a statistically significant difference at 10 dilution factor from “no addition” (*p* = 3.3 × 10^−4^) (Figure 7a), none of the three anti-cSCS antibodies inhibited the binding of spike to its receptor (ACE2) in vitro (*p* = 0.30, *p* = 0.22, and *p* = 0.12 for Peptides 1, 2, and 3, respectively) (Figure 7b–d). These antibodies were not neutralizing antibodies. The commercial anti-spike antibody slightly inhibited the binding of spike to ACE2, but the difference was not statistically significant (*p* = 0.055) (Figure 7e). The commercially available anti-IL-7 antibody did not inhibit the binding of the spike protein to ACE2 (*p* = 0.20) (Figure 7f).

**Figure 8:**
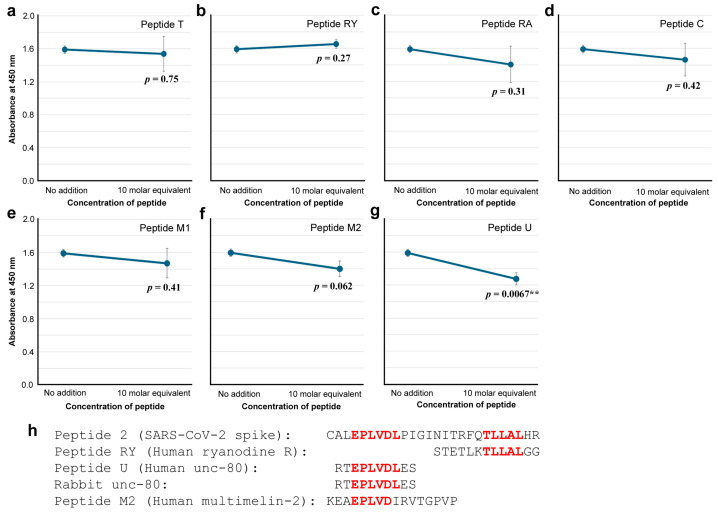
ELISA for anti-Peptide 2 antibody binding to the spike protein in the presence of a human-derived peptide (ELISA #3 in Figure 1). The indicated *p*-values were obtained by two-sided unpaired Student’s *t* tests between the sample with the peptide concentration tested (10 molar equivalent) and the sample with “no addition” of a peptide (control). The error bars indicate the standard deviation. Asterisks indicate statistical significance (**: *p* < 0.01). The molar equivalent was calculated by assuming that the molecular weight of IgG is 150 kDa. (**a**) Peptide T. (**b**) Peptide RY. (**c**) Peptide RA. (**d**) Peptide C. (**e**) Peptide M1. (**f**) Peptide M2. (**g**) Peptide U. (**h**) Important peptide and protein sequences, with cSCSs shown in red.

To further characterize the anti-Peptide 2 antibody, seven peptides that have human protein sequences containing cSCSs were synthesized and tested for their ability to competitively inhibit the binding of the anti-Peptide 2 antibody to the spike protein (Figure 8a–g). Among them, peptides T (*p* = 0.75), RY (*p* = 0.27), RA (*p* = 0.31), C (*p* = 0.42), M1 (*p* = 0.41), and M2 (*p* = 0.062) did not show a statistically significant difference. Only peptide U from human unc-80 significantly inhibited the binding of the anti-Peptide 2 antibody (*p* = 0.0067) (Figure 8g). Peptide M2 seemed to inhibit the binding to some extent but not significantly (*p* = 0.062) (Figure 8f). Sequence comparisons among important peptides (Figure 8h) revealed that only EPLVDL was common between Peptides 2 and U, suggesting that the anti-Peptide 2 antibody recognized EPLVDL in both the spike protein and the human unc-80 protein. The entire Peptide U sequence was found not only in human unc-80 but also in rabbit unc-80 (Figure 8h), suggesting that the rabbit immunized with Peptide 2 produced the anti-Peptide 2 antibody regardless of the presence of the self-epitope EPLVDL in the rabbit unc-80.

### 3.5. Western Blot Analysis

**Figure 9:**
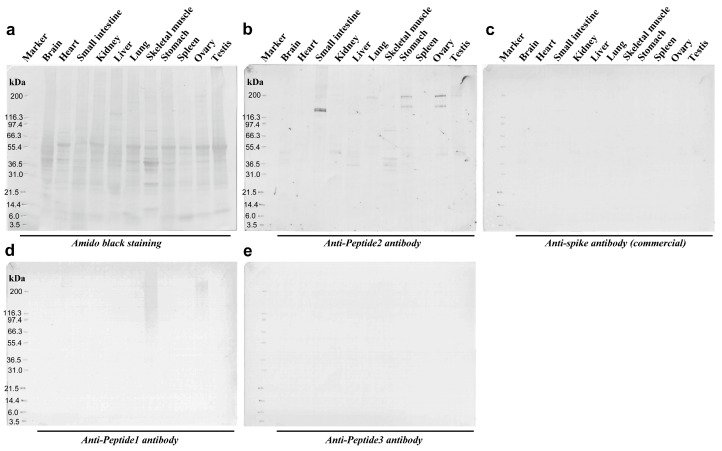
Western blot analyses of anti-spike antibodies. (**a**) Amido black staining confirm that the proteins were successfully blotted on the membrane. (**b**) Anti-Peptide 2 antibody. Intense signals were observed in the small intestine. Less intense signals were observed in the ovary, stomach, skeletal muscle, and other various tissues. (**c**) Anti-spike antibody (commercial). No signal was observed. (**d**) Anti-Peptide 1 antibody. Smear bands were observed in the ovary and skeletal muscles. (**e**) Anti-Peptide 3 antibody. No signal was observed. The original Western blot figures can be found in Figure S1.

We tested whether the anti-Peptide 2 antibody and other antibodies cross-reacted with human proteins via Western blot analysis. We used a membrane on which human proteins from 11 organs were blotted, as revealed by amido black staining (Figure 9a). The anti-Peptide 2 antibody strongly stained the band at approximately 140 kDa from the small intestine as a double band (Figure 9b). This antibody also stained bands of higher molecular weights from the stomach and ovary (160 kDa and 200 kDa) and other small and weaker bands from various organs (Figure 9b). In contrast, the commercially available anti-spike antibody did not stain any protein (Figure 9c). The anti-Peptide 1 antibody stained smear bands from the ovary and skeletal muscle at approximately 200 kDa (Figure 9d). The anti-Peptide 3 antibody did not stain any protein (Figure 9e).

## 4. Discussion

### 4.1. High Occurrence of cSCSs Is Likely a Coincidence

Our exhaustive in silico analyses of the human proteome suggested that cSCS distributions in human proteins are not particularly biased biologically (Figure 2a–f). They showed a very weak length-dependent distribution in proteins. The cSCS frequency is more or less similar among proteins of various lengths, but shorter proteins may have a slightly greater frequency (density) of cSCSs. We speculate that this result is simply due to more repeat sequences (and hence less diverse sequences) in longer proteins. On the other hand, we hypothesized that cytokines may be potential targets of cSCSs for autoantibodies partly because they are generally small proteins. However, we did not detect any bioinformatics evidence that cytokines contain more cSCSs than other proteins.

We also showed that the cSCS distribution followed a power law distribution, similarly to the Zipf distribution (Figure 2b), suggesting a language-like stochastic pattern [79,80]. Again, we did not detect any biological bias. Similarly, according to GO annotations, proteins in the immune system did not contain more cSCSs than proteins in other functional categories. Rather, cSCSs seemed to be distributed almost evenly among functional categories of proteins (Figure 2g,h). Therefore, we conclude that the high occurrence of cSCSs between the SARS-CoV-2 spike protein and the human proteome is not biological but likely coincidental. This conclusion is consistent with the assumption that SARS-CoV-2 did not have any evolutionary history with humans before December 2019. Most likely, we speculate that many other viral proteins would show a similarly high occurrence of cSCSs in humans without any evolutionary interactions.

In this sense, it may be misleading to call the high occurrence molecular mimicry because, in biology, “mimicry” implies a product of evolution to avoid being attacked by predators, as originally proposed in butterflies [45]. To discuss short constituent sequences (or “peptides”) in proteins, molecular “commonality” or “coincidence” rather than mimicry, similarity, or homology is probably a better term. Molecular similarity and homology are not accurate in the SCS analysis because cSCSs in SARS-CoV-2 and humans are not similar or homologous but are identical. The use of the term “peptide” instead of a short constituent sequence (SCS) is also often confusing because a part of the protein sequence is not a peptide unless it is isolated chemically. In any case, the conventional concept of the molecular mimicry theory in the context of immunology is simply to indicate the production of antiviral antibodies that cross-react with human proteins, which we evaluated in this study in silico and in vitro.

To be sure, spike epitopes have been reported in COVID-19 patients [65,66,67,68,69,70,71,72,73,74,75,76], and there are many spike epitopes that have been reported in the Immune Epitope Database (IEDB) [110,111,112], in which Peptides 1, 2, and 3 are all well covered. We found that most cSCSs were not located on the surface of proteins in the extracellular milieu, that is, most cSCSs cannot function as epitopes until they are exposed to protein degradation or conformational changes. By analyzing three-dimensional structures and solvent accessibility scores, we focused on six candidate cSCSs for further in vitro studies that may function as epitopes for autoantibodies (Figure 3; Table 1). We believe that most cSCSs in SARS-CoV-2 cannot be considered a threat to molecular mimicry for autoimmunity. Nevertheless, this does not mean that all cSCSs are safe. A small number of anti-spike antibodies may be harmful, as shown in the present study. Furthermore, some cSCSs located deep in a protein molecule may be exposed following conformational changes and degradation. The exposed cSCS fragments may be released into the blood stream, for example, upon hyperinflammation in COVID-19 [118,119,120]. Because vaccines are administered to healthy people, safety issues related to vaccines cannot be overemphasized.

### 4.2. Anti-Peptide 2 Antibody Is an Autoantibody

Our strategy to use 5-aa cSCSs as a unit of sequence analysis appears to be successful not only in silico but also in vitro because they served as functional epitopes for antibodies, as expected. Stretches of amino acid sequences that are longer than five amino acids were also found as consecutive cSCSs. The anti-Peptide 2 antibody was shown to recognize a six amino acid cluster of cSCSs (EPLVDL) of the SARS-CoV-2 spike protein and Peptide 2 itself (Figure 5 and Figure 6). The anti-Peptide 2 antibody was also shown to recognize Peptide U, derived from the human unc-80 protein (Figure 8). The anti-Peptide 2 antibody was not a neutralizing antibody in vitro (Figure 7). Considering that most neutralizing antibodies target the RBD of spike [65,66,67,68,69,70,71,72,73,74,75,76], this result may not be surprising because the Peptide 2 sequence is located outside the RBD (Figure 4). Moreover, a commercial polyclonal anti-spike antibody did not clearly show neutralizing characteristics. Only a portion of this polyclonal antibody against some specific epitopes may have a neutralizing ability.

The anti-Peptide 3 antibody did not bind to the spike protein well (Figure 5). Thus, the production of an anti-Peptide 3 antibody may be considered a simple technical failure. Similarly, an anti-IL-7 antibody did not cross-react with the spike protein (Figure 5). This is unexpected, considering that IL-7 has three consecutive cSCSs (KLNDL, LNDLC, and NDLCF) as a seven amino acid cluster. A general conclusion here would be that cytokines, including IL-7, do not seem to be off-targets of anti-spike antibodies. However, we cannot exclude the possibility that different antibodies against IL-7 may result in positive ELISA results. Further studies on IL-7 may be expected in the future. Considering that we used polyclonal antibodies in this study, the use of monoclonal antibodies may clarify these issues associated with antibody characteristics.

Interestingly, the secondary structure of EPLVDL varies; it is a β-strand in spike and a fusion of an α-helix and a loop in unc-80 (Figure 3), but it should be noted that these secondary structures do not seem to matter much for antibody recognition, considering that antibodies can be raised against peptides (likely not structured) and that antibodies can recognize denatured proteins on a membrane in Western blot analysis. In any case, the available data reasonably suggest that the anti-Peptide 2 antibody is an autoantibody against EPLVDL that is present both in the SARS-CoV-2 spike protein and in the human unc-80 protein.

### 4.3. The Human Unc-80 Protein Is a Potential Target of an Autoantibody

We were not able to show that the anti-Peptide 2 antibody could directly recognize an intact unc-80 protein in this study. The identities of the positive bands detected with the anti-Peptide 2 antibody on the Western blot membrane at approximately 140, 160, and 200 kDa (Figure 9) are not clear because the human unc-80 protein and ryanodine receptor have different predicted molecular weights of 363 and 63 kDa, respectively. There is the possibility that the multiple bands may be due to the polyclonality of the anti-Peptide 2 antibody; the use of monoclonal antibodies may resolve this issue. Smear bands detected with the anti-Peptide 1 antibody at approximately 200 kDa are not clear because Peptide 1 was derived from teneurin-4, and its molecular weight is 442.1 kDa. However, the Western-positive bands may be fragments of these proteins. The anti-Peptide 3 antibody did not show any positive bands. The Peptide 3 antibody was raised against a sequence of IL-7, 17.5 kDa, which was covered with the Western membrane used in this study. These Western blot results may be largely consistent with the ELISA results.

Therefore, we conclude that the anti-Peptide 2 antibody is likely an autoantibody when it is produced in humans in vivo. The anti-Peptide 1 antibody may also be an autoantibody in vivo with relatively low affinity for spike and human proteins, although its ELISA and Western blot results were not very convincing. The anti-Peptide 3 antibody does not seem to function as an autoantibody, even when it is produced in humans in vivo. Somewhat surprisingly, a commercial anti-spike antibody did not stain any band in the Western blot analysis. This could simply be because this anti-spike antibody is polyclonal against different epitopes of the spike protein. It should also be noted that the Western blot analysis detects denatured proteins on the membrane. The possibility remains that the Western-negative antibodies can recognize conformational epitopes in native proteins. However, these antibodies were raised against short peptides, which may not be stably folded into three-dimensional structures. In any case, immunoprecipitation may be a way to further characterize these antibodies and identify human proteins interacting with them.

Although uncertain at this point, the human protein that anti-Peptide 2 antibodies may recognize could be unc-80, and if so, its functional inhibition by such autoantibodies in COVID-19 patients and vaccinated populations may be a medical and social concern. Unc-80 is a part of the NALCN channel complex that regulates the resting membrane potential in excitable cells [121,122]. The channel complex, which includes unc-80, is activated by peptide neurotransmitters to contribute to slow synaptic responses [123]. The NALCN complex regulates neuronal excitability via calcium influx [124]. Unc-80 mutations potentially cause a wide variety of neural disorders, including schizophrenia, Alzheimer’s disease, autism, and cognitive delay [125]. Moreover, unc-80 mutations cause severe disorders such as hypotonia and intellectual disability [125,126]. According to a bioinformatics study, unc-80 has been suggested to be a hub gene for pancreatic cancer [127]. NALCN plays an important role in pacemaker activity [128], sensation, and pain [129]. We speculate that the presence of anti-spike antibodies that cross-react with unc-80 may partly explain the diverse range of symptoms in patients with COVID-19 and long COVID.

### 4.4. Implications for Vaccine Development, Long COVID, Tolerance, and Memory

Although only a small number of autoepitope candidates, among many others, appear to be functional autoepitopes, the present study demonstrated the possibility that SARS-CoV-2 infection and vaccination induce autoantibodies that may be harmful to patients. Here, we focused on human proteins that have five or more cSCSs, but we did not exclude the possibility that a single cSCS could function as an efficient epitope. In this sense, more cSCSs should be examined in vitro for their antigenicity in the future. Based on the present results, we propose that the EPLVDL sequence in spike-based vaccines should be changed to “benign” uncommon SCSs to avoid a potential risk of autoimmunity. The anti-EPLVDL antibody, if produced, does not seem to work against infection because it is unlikely to be a neutralizing antibody, at least in vitro.

It is known that infection with SARS-CoV-2 induces various autoantibodies and autoimmune diseases [65,66,67,68,69,70,71,72,73,74,75,76]. These autoantibodies do not seem to directly target the SARS-CoV-2 spike protein. Thus, molecular mimicry in the conventional sense may not directly contribute to such autoimmunity. Nevertheless, cSCSs such as EPLVDL may induce anti-cSCS antibodies in COVID-19 patients, which may then trigger systemic production of many kinds of autoantibodies (but not against SARS-CoV-2) by lowering the general threshold for self/nonself discrimination. In this sense, the present study may shed light on mechanisms of immunological tolerance or self/nonself discrimination.

There is a possibility that B-cell clones that produce antibodies against EPLVDL are forbidden from surviving by regulatory T cells to prevent the production of autoantibodies. Contrary to this possibility, the anti-cSCS antibody was produced in rabbits despite EPLVDL also being found in the rabbit unc-80 protein, suggesting that such B-cell clones may be allowed to survive and produce antibodies at least upon challenge with the EPLVDL antigen in rabbits (Figure 8h). The immunological threshold for tolerance may change in response to the relative amount of antigens in the body. It is understood that self-destruction by autoantibodies compensates for the acute defense of the body from the virus. If such autoantibodies are continuously produced in patients even after the clearance of the acute phase, long COVID may persist. Under such circumstances, memory B cells that produce autoantibodies against cSCSs should be eliminated smoothly once the acute phase of infection is cleared. This is not to harm its own body. We speculate that this may be why a single infection or vaccination is not enough to form long-term immunological memory in the case of SARS-CoV-2 and other viruses. If this line of argument is correct, repeated vaccinations against SARS-CoV-2 should be limited to specific populations to avoid the negative long-term effects of autoimmunity in the public. To augment or supplement the vaccine strategy, other modes of interventions, such as nitric oxide [130,131] and drugs [132,133,134,135], may also be considered in a positive manner together with vaccination.

## 5. Conclusions

This study demonstrated the validity of an SCS-based strategy in the search for common sequences between two organismal systems. The present results suggest that cSCSs do not seem to exhibit “mimicry” in a biological sense. Rather, cSCSs are likely coincidental, and most cSCSs are likely negative for autoantibody production. Nevertheless, it is noteworthy that a stretch of the amino acid sequence, EPLVDL, is the epitope for the anti-Peptide 2 antibody that recognizes not only the SARS-CoV-2 spike protein but also human proteins, probably including unc-80. The present study highlights the potential risk of current SARS-CoV-2 vaccines that use the “raw” spike sequence as a large epitope sequence. We propose manufacturing safer vaccines by modifying EPLVDL and other potentially risky cSCSs in the spike protein.

## Figures and Tables

**Table 1:** cSCSs located on the surface of human proteins with their accessibility scores.

Human Protein	cSCS(S1/S2)	Accessibility (Method I) *		Accessibility (Method II) *	
		Spike	Human Protein	Spike	Human Protein
Ryanodine receptor 1 Ryanodine receptor 2 Ryanodine receptor 3	TLLAL (S1)	003XX	00243	10110	10130
Protein unc-80 homolog	EPLVD (S1)	20633	XXXXX	21102	44435
	PLVDL (S1)	06330	XXXXX	11020	44354
	LPDPS (S2)	02388	XXXXX	02435	14544
Teneurin-4	GTTLD (S1)	02513	30547	12214	12435
Neural adhesion molecule L1-like protein	PSKPS (S2)	885X5	29543	35545	35312
IL-7	KLNDL (S1)	20250	74563	31242	45542
	LNDLC (S1)	02501	45630	12421	55420
	NDLCF (S1)	25010	56303	24211	54202
	LVLLP (S1)	XXXXX	31550	00001	00001

* Accessibility was evaluated by two methods: Methods I and II (see Section 2).

## Data Availability

All data are available in this paper and its associated Supplementary Materials.

## Supplementary Materials

The following supporting information can be downloaded at https://www.mdpi.com/article/10.3390/vaccines12050539/s1, Figure S1: Original pictures for Figure 9; Supplementary File S1: “SARS-CoV-2 Spike vs. Human Original Text” (text file); Supplementary File S2: “Spike vs. Human Excel #1” (Excel file); Supplementary File S3: “Spike vs. Human Excel #2” (Excel file); Supplementary File S4: “Excel Programming for Informatics” (PDF file).
