# Supplementary material for: In Silico and In Vitro Evaluation of the Molecular Mimicry of the SARS-CoV-2 Spike Protein by Common Short Constituent Sequences (cSCSs) in the Human Proteome: Toward Safer Epitope Design for Vaccine Development
Source: Vaccines (Basel). 2024 May 14;12(5):539. doi: 10.3390/vaccines12050539 (PMC11125730; doi:10.3390/vaccines12050539)
Supplement: Supplementary file 1 [file vaccines-12-00539-s001.zip › Figure S1 Original pictures for Figure 9.pdf]

a

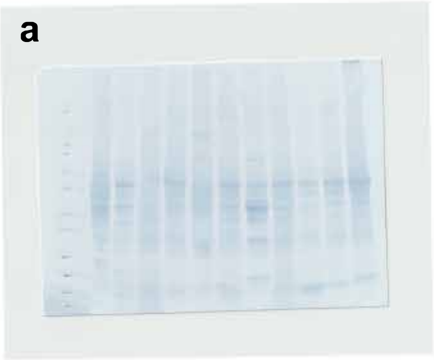

Amido black

b

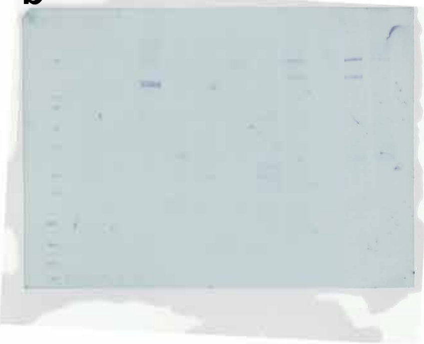

Anti-P2 antibody

c

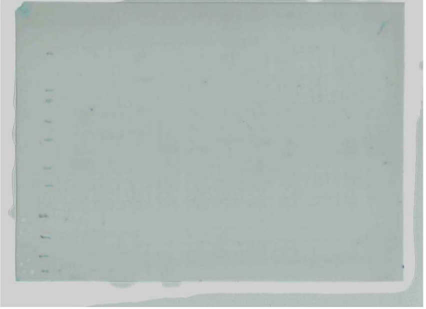

Anti-SARS-CoV-2 antibody
(Commericial)

d

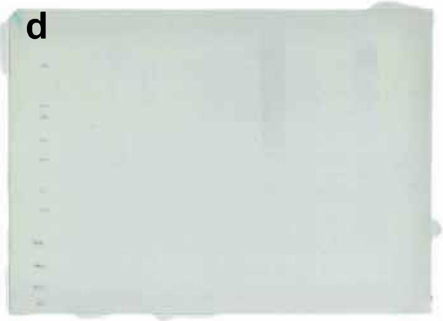

Anti-P1 antibody

e

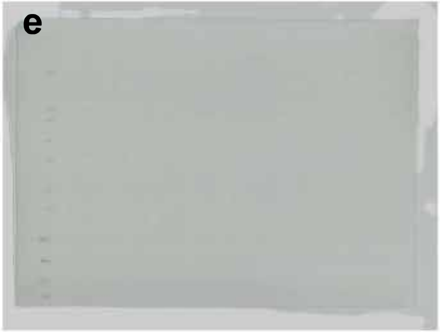

Anti-P3 antibody

Figure S1. Original pictures for Figure 9.
