# Supplementary material for: In Silico and In Vitro Evaluation of the Molecular Mimicry of the SARS-CoV-2 Spike Protein by Common Short Constituent Sequences (cSCSs) in the Human Proteome: Toward Safer Epitope Design for Vaccine Development
Source: Vaccines (Basel). 2024 May 14;12(5):539. doi: 10.3390/vaccines12050539 (PMC11125730; doi:10.3390/vaccines12050539)

Supplementary File S4. Excel Programming For Informatics

The following two files were used in the steps ①-⑨ below.

Supplementary File S1. SARS-CoV-2 Spike vs. Human Original

Text **Supplementary File S2.** Spike vs. Human Excel #1

① All data from Supplementary File S1 were copied and pasted on an Excel file to make Supplementary File S2 (Sheet 1).

② Only cells with protein names were extracted. Using the autofilter function, strings containing "Homo sapiens" in the column A of Sheet 1 were extracted and were placed in the column A of Sheet 2 from the top (Sheet 2).

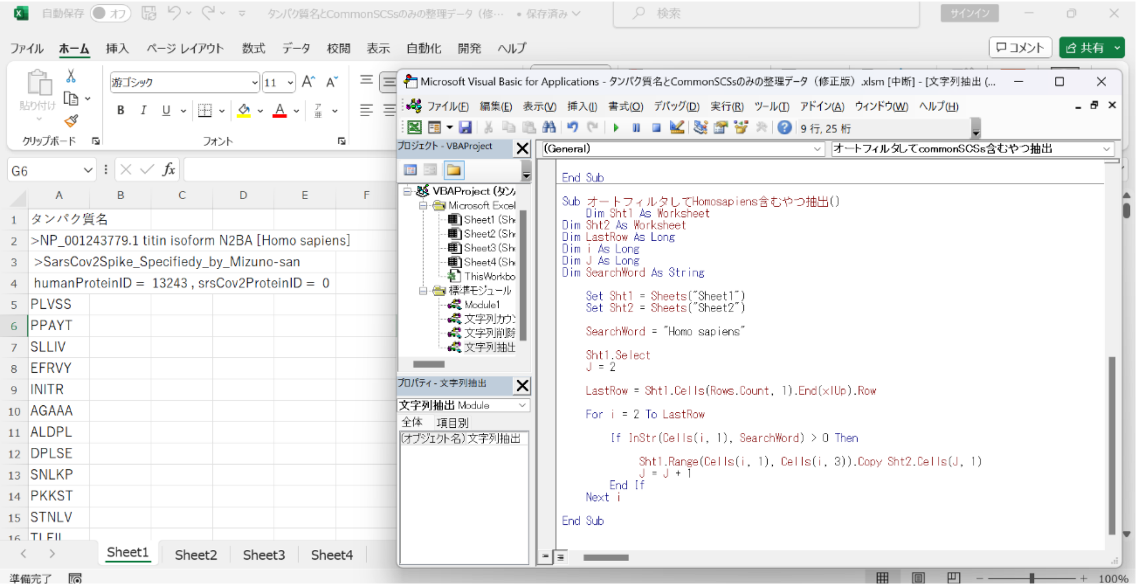

③ Only cells with "commonSCSs" (cSCSs) were extracted. Similarly, using the autofilter function, strings containing "commonSCSs" in the column A of Sheet 1 were extracted and were placed in the column B of Sheet 2 from the top (Sheet 2).

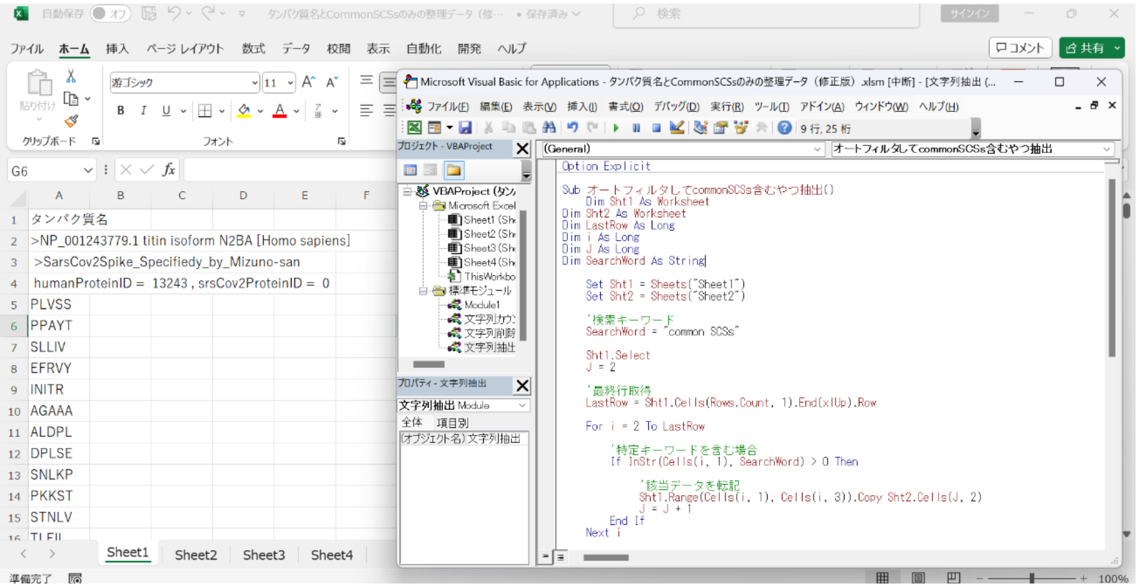

④ Strings containing "Homo sapiens" and "CommonSCSs = " were deleted. Using the Replace function, the data after deleting strings of "Homo sapiens" from the column A of Sheet 2 were placed from the top. Using the Right function, regarding 1st-40th lines of the column B of Sheet 2, two digits from the right were extracted by a program. Similarly, regarding 41st-last lines, one digit from the right were extracted by another program. Only the numbers of cSCSs were placed in the column E of Sheet 2.

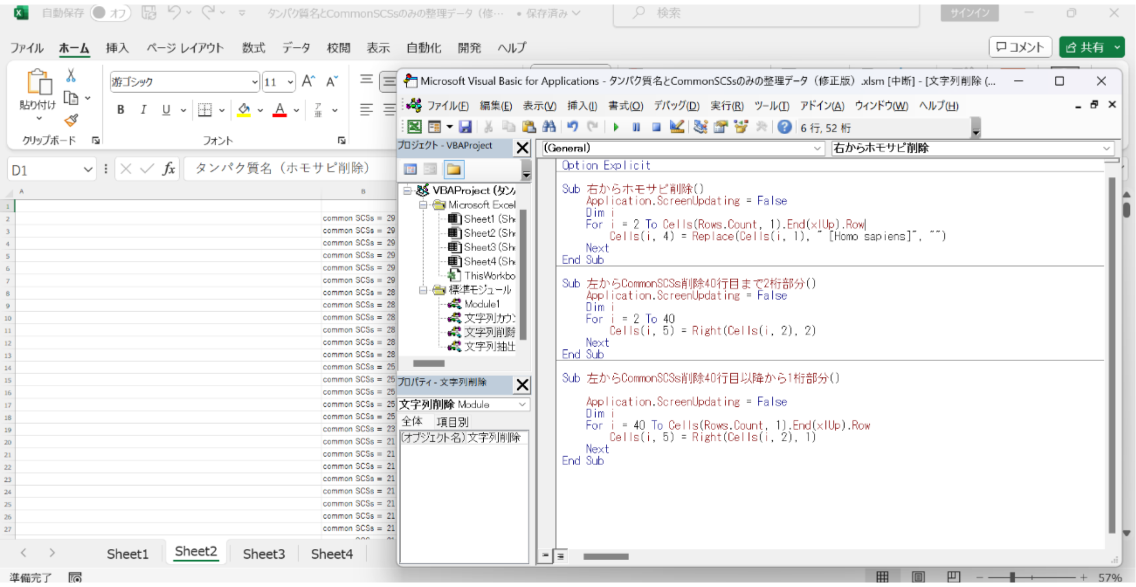

⑤ To delete strings in front of protein names, using the Mid function, strings after protein names were extracted from the strings in the column D of Sheet 2.

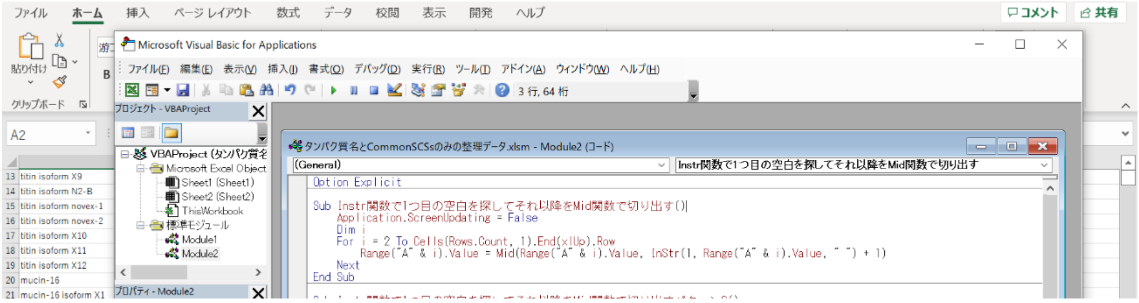

⑥ To delete strings "isoform" in front of protein names, the Left function was used. The data were copied and pasted in the column A of Sheet 3.

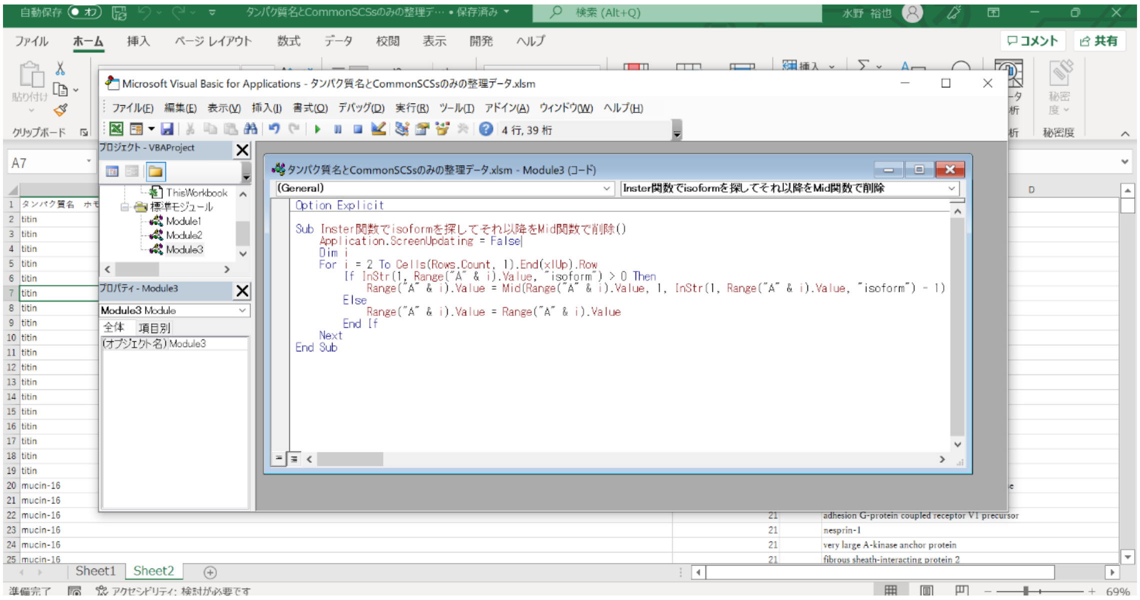

⑦ To delete redundant entry of protein names due to isoforms in the column A of Sheet 3, using the AdvancedFilter function, nonredundant protein names were placed in the column B of Sheet 3 from the top.

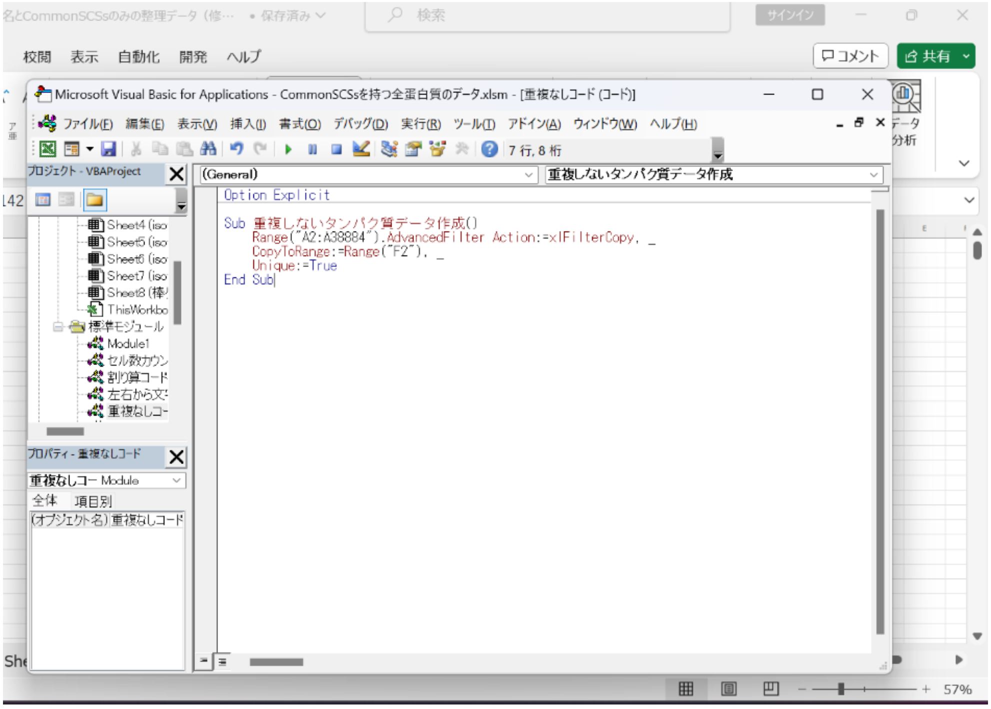

⑧ To count the numbers of isoforms, regarding proteins with isoforms, using the countIF function, the numbers of protein names being present in the column A of Sheet 3 were counted. The data were placed in the column C of Sheet 3.

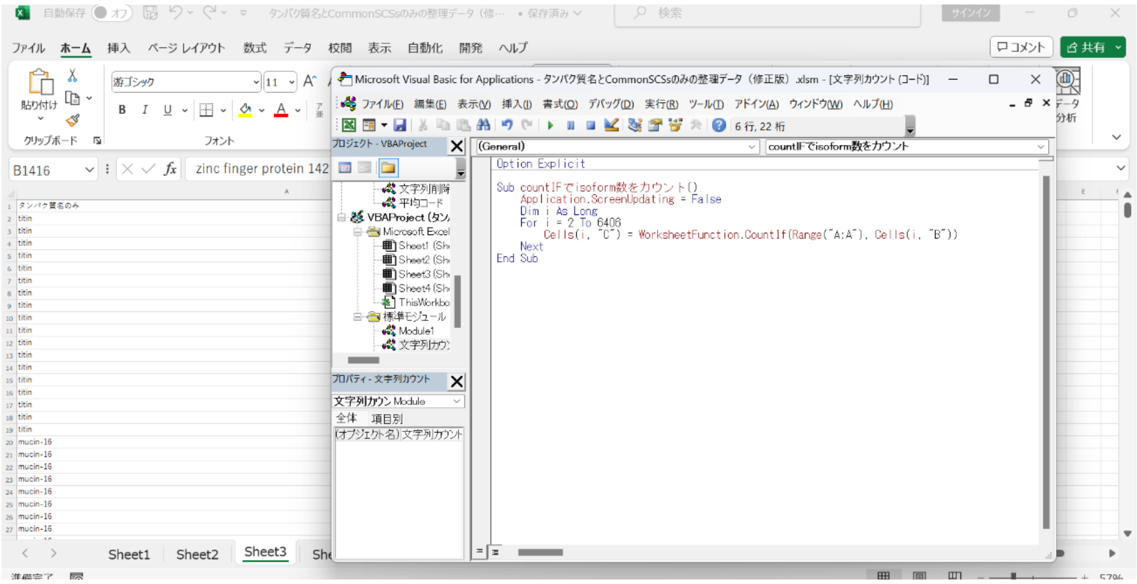

⑨ **Making association of the numbers of amino acids (An independent step being different from the steps above).** In Sheet 4, the numbers of amino acids that were obtained from NCBI were placed. These data were made through the extraction of the numbers of amino acids that were associated with the protein data from NCBI when the original text file data were made. The extracted data were placed in the column B, and protein names were

placed in the column, to indicate their pairs. Among these data, there were some entries in which only the numbers in the Y portion of “>XX_YYYYYYYYYY” were different, although protein names and isoform numbers were identical. (e.g., zinc finger and SCAN domain-containing protein 26 isoform X4). Thus, these protein entries should be treated as single entries. To do so, according to the step ⑧ above, using the AdvancedFilter function, nonredundant protein names were placed in the column C from the tip, and their corresponding numbers of amino acids were placed in the column D, using the Vlookup function.

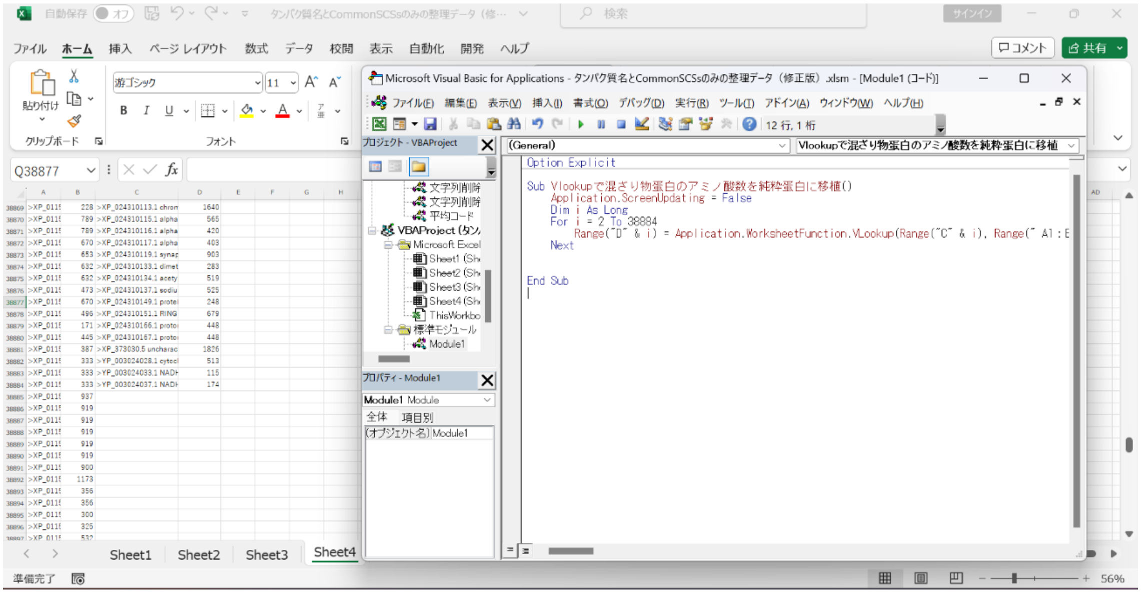

The following file was used in the steps ⑩-⑬ below.

Supplementary File S3. Spike vs. Human Excel #2

⑩ The completed data through the steps above were copied and pasted in the Sheet “The most important data”. Using these data, calculations were performed, according to the steps below.

⑪ For all proteins, we calculated cSCSs/amino acid. To do so, the column B was divided by the column C.

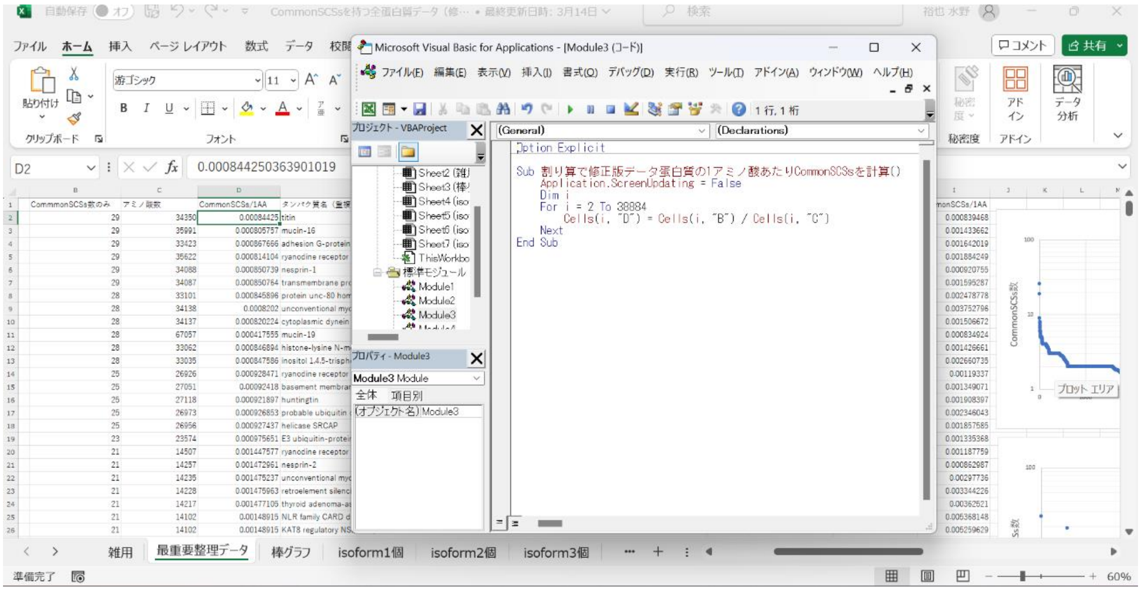

⑫ We calculated the average numbers of cSCSs in proteins. To do so, using the AverageIF function, from the range of the columns A and B, the average numbers of cSCS were calculated for proteins in the column E from the top, and the data were placed in the column G alongside the corresponding protein names.

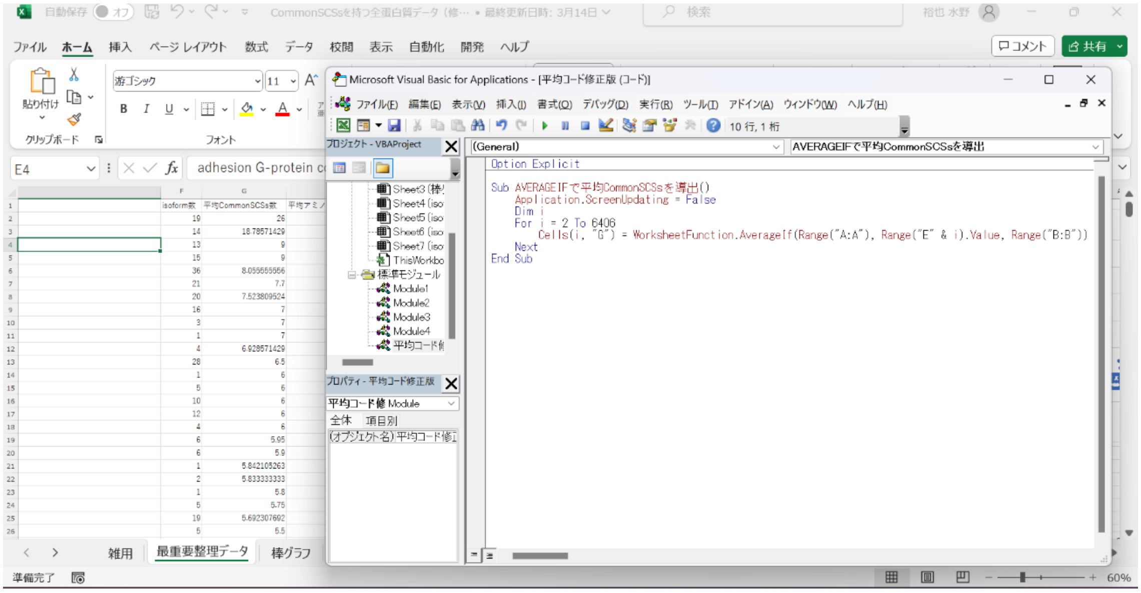

⑬ We calculated the average numbers of amino acids in proteins and the average numbers of cSCSs/amino acid. To do so, according to the step ⑪ above, using the AverageIf function, the average numbers of amino acids were placed in the column H, and the average numbers of cSCS/amino acids were placed in the column I.

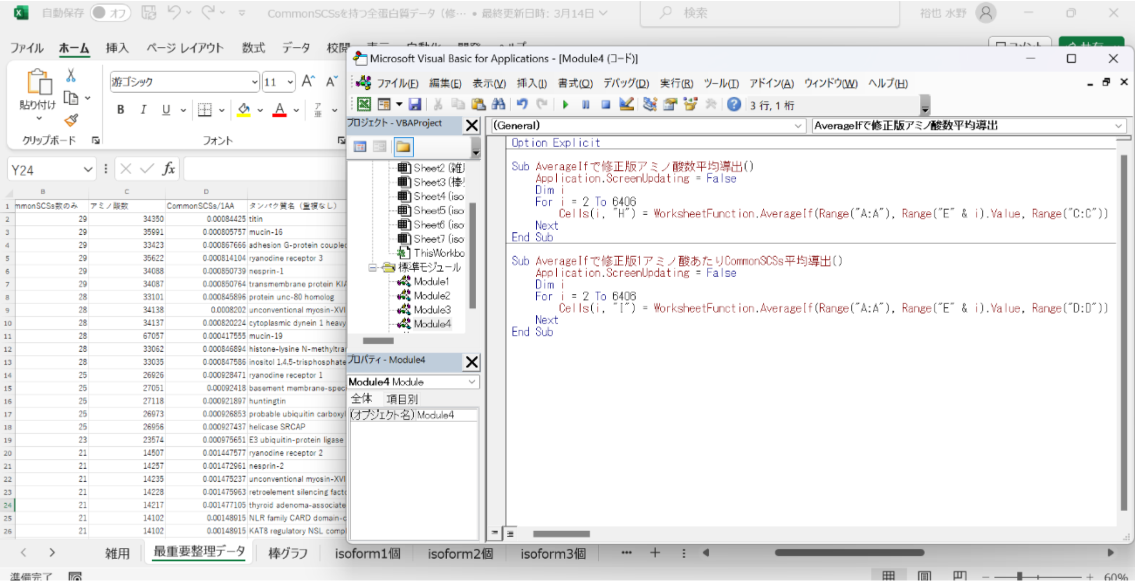

Supplement: Supplementary file 1 [file vaccines-12-00539-s001.zip › Supplementary File S4 Excel Programming for Informatics.pdf]
